# The influence of changes in trunk and pelvic posture during single leg standing on hip and thigh muscle activation in a pain free population

**DOI:** 10.1186/2052-1847-6-13

**Published:** 2014-03-27

**Authors:** Simon Prior, Tim Mitchell, Rod Whiteley, Peter O’Sullivan, Benjamin K Williams, Sebastien Racinais, Abdulaziz Farooq

**Affiliations:** 1 Lennox Head Physiotherapy Centre, 48 Ballina St, Lennox Head 2478, NSW, Australia; 2 Curtin University, Perth, WA, Australia; 3 ASPETAR - Qatar Orthopaedic and Sports Medicine Hospital, Doha, Qatar; 4 Aspire Academy, Doha, Qatar; 5 Athlete Health and Performance Research Centre, Aspetar, Qatar Orthopaedic and Sports Medicine Hospital, Doha, Qatar

**Keywords:** Single leg stance, Trunk and pelvis posture, EMG, Motor patterns, Joint position, Groin

## Abstract

**Background:**

Thigh muscle injuries commonly occur during single leg loading tasks and patterns of muscle activation are thought to contribute to these injuries. The influence trunk and pelvis posture has on hip and thigh muscle activation during single leg stance is unknown and was investigated in a pain free population to determine if changes in body posture result in consistent patterns of changes in muscle activation.

**Methods:**

Hip and thigh muscle activation patterns were compared in 22 asymptomatic, male subjects (20–45 years old) in paired functionally relevant single leg standing test postures: Anterior vs. Posterior Trunk Sway; Anterior vs. Posterior Pelvic Rotation; Left vs. Right Trunk Shift; and Pelvic Drop vs. Raise. Surface EMG was collected from eight hip and thigh muscles calculating Root Mean Square. EMG was normalized to an “upright standing” reference posture. Repeated measures ANOVA was performed along with associated F tests to determine if there were significant differences in muscle activation between paired test postures.

**Results:**

In right leg stance, Anterior Trunk Sway (compared to Posterior Sway) increased activity in posterior sagittal plane muscles, with a concurrent deactivation of anterior sagittal plane muscles (p: 0.016 - <0.001). Lateral hip abductor muscles increased activation during Left Trunk Shift (compared to Right) (p :≤ 0.001). Lateral Pelvic Drop (compared to Raise) decreased activity in hip abductors and increased hamstring, adductor longus and vastus lateralis activity (p: 0.037 - <0.001).

**Conclusion:**

Changes in both trunk and pelvic posture during single leg stance generally resulted in large, predictable changes in hip and thigh muscle activation in asymptomatic young males. Changes in trunk position in the sagittal plane and pelvis position in the frontal plane had the greatest effect on muscle activation. Investigation of these activation patterns in clinical populations such as hip and thigh muscle injuries may provide important insights into injury mechanisms and inform rehabilitation strategies.

## Background

Thigh muscle injuries including the hamstring and adductor groups account for a large proportion of missed training and playing time in sports such as soccer, football and sprinting [1,2]. There is some evidence to support that altered muscle function during single leg loading may be a contributing factor in hamstring muscle strains [3], and athletic groin pain [4,5]. however the mechanisms behind this altered muscle function are not clear [6-8].

In addition, retraining of hip and thigh muscle groups as part of prevention and rehabilitation of these thigh muscle injuries is popular, with a huge variety of exercises and exercise parameters being recommended [9-11]. There is growing support for functional retraining as an important component of injury prevention and rehabilitation strategies, however there remains a lack of understanding regarding factors that strongly influence muscle function during single leg loading. We hypothesized that position of the trunk and pelvis during single leg loading strongly influences the activation patterns of the hip and thigh muscles. To date, a number of studies investigating frontal plane pelvis position (pelvic drop or Trendelenberg posture) in single leg loading show pelvic posture does influence activity of the hip abductor muscles [12-14].

Apart from these studies, there is little evidence regarding how changes in trunk and pelvis position influence muscle activation patterns in common fontal and sagittal plane postures in single leg stance. There is some literature to suggest that changing posture in a sagittal plane whilst in double leg stance changes the activation of different muscles. O’Sullivan and co-workers [15] demonstrated differences in abdominal and back muscle activity levels when comparing active upright standing to posterior trunk sway standing. However only trunk, not hip and thigh muscle activity was recorded in this study. This knowledge has lead to improved understanding of potential pain mechanisms linked to standing posture [16] and functional rehabilitation strategies for back pain disorders [17].

Wang and co-workers [18] showed that with anterior trunk sway, there was an increase in hamstring and erector spinae activation (dorsal muscles), accompanied by a decrease in rectus femoris and rectus abdominus activation (ventral muscles), with the opposite pattern observed in posterior trunk sway. Neither study evaluated single leg loading. Other studies have shown that altering lower limb or hip position during single leg loading influences hip and thigh muscle activation [19-21] however the influence of more proximal body segment posture on muscle activity has not been investigated.

In summary, despite what would appear to have widespread clinical application, the influence that trunk and pelvis posture has on lower limb muscle activation in single leg stance is largely unknown. The aim of this study was to investigate the influence of changes in frontal and sagittal plane positions of the trunk and pelvis on muscle activation around the hip and thigh in single leg stance in a male pain free population.

It was hypothesized that changes in both trunk and pelvic posture during single leg stance would result in predictable changes in muscle activation. Specifically, changing posture in the frontal plane would alter primarily frontal plane muscle activity, and changes of posture in the sagittal plane would alter primarily sagittal plane muscle activity.

## Methods

### Participants

Twenty two asymptomatic, male subjects aged between 20–45 years old were recruited via personal invitation and gave written informed consent to participate ensuring the rights of each subject were protected. Ethical approval was granted by the Human Research Ethics Committee of Curtin University of Technology (approval number: HR 25/2011), Perth, Australia and Aspetar Sports Medicine Hospital, Doha, Qatar. Testing took place in the biomechanical laboratories of Aspire Sports Academy, Doha, Qatar.

As body mass index (BMI) has been shown to influence EMG amplitude [22] subjects were excluded if their BMI > 30. Subjects were also excluded if they: had a lower limb or back injury within the last three months that had restricted participation in their usual physical activities; or were unable to adopt and sustain the required test postures. An a priori power analysis showed that twenty subjects were required to achieve a significant difference in EMG with an alpha level of 0.05 and 80% power; accordingly 22 were recruited to allow for data loss.

### Test postures

3D Kinematic data was monitored using a 14 camera Vicon (OMG, England), Full Body Plug-in Gait model (OMG, England) (excluding upper limb and head markers), with MX-13 cameras (OMG, England) through Vicon Nexus software (OMG, England), at a sampling rate of 500 Hz.

**Figure 1:**
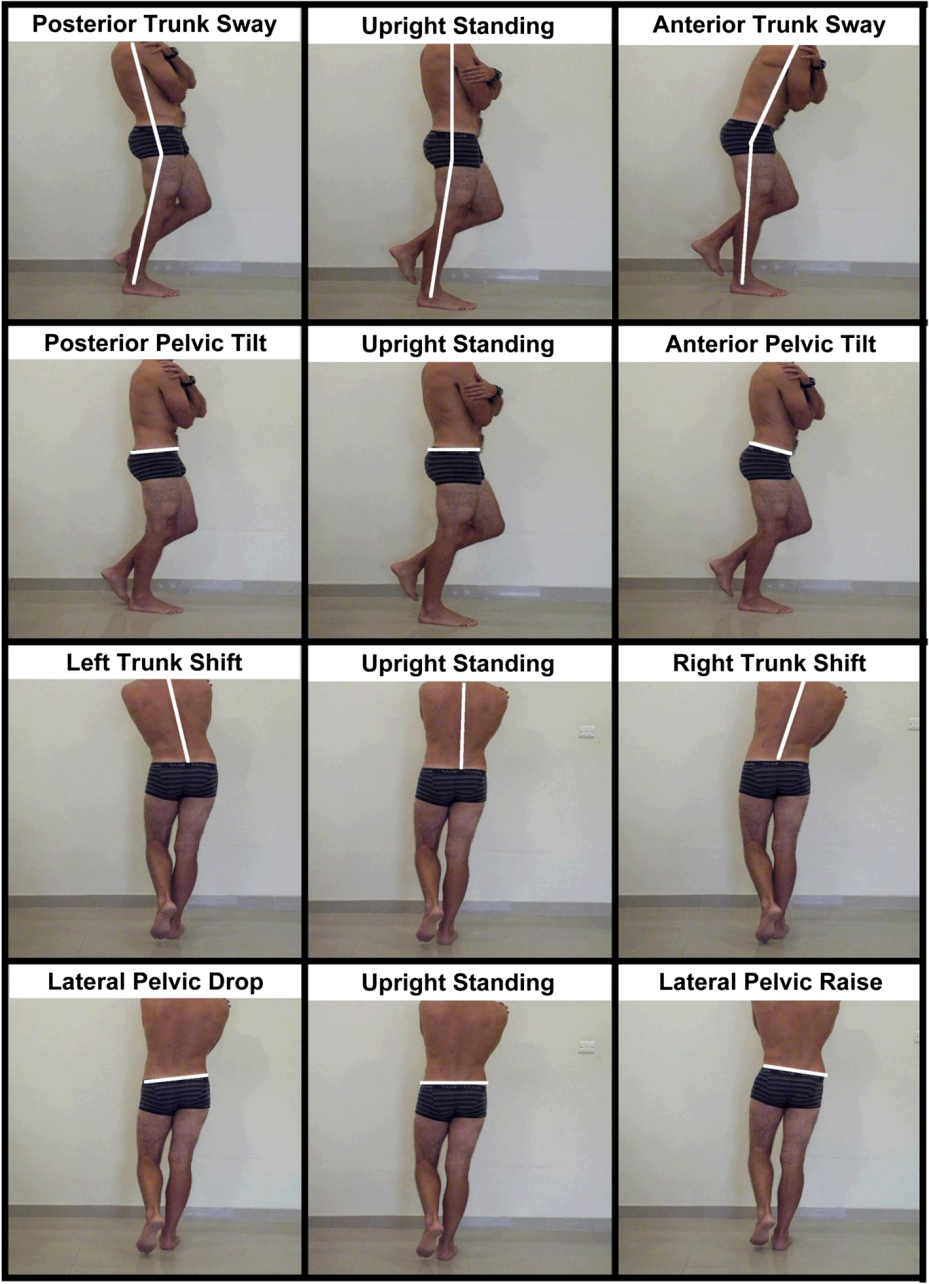
Depictions of each of the 4 pair wise comparison positions with the reference upright standing position.

4 pairs of common functional trunk and pelvic positions were tested. All test postures were defined relative to a reference single leg “Upright Standing” posture (Figure 1).

### Upright standing

Upright Standing was defined as a position in which the subject stood on the right leg with the right acromion, right greater trochanter, and right lateral malleolus vertically aligned (+/− 10°). The subject was instructed to unlock the right knee in slight (approximately 10°) flexion. For each test posture, subjects stood on their right bare foot, arms folded, head stable and eyes looking forward at a fixed point. Each testing session was carried out by the same investigator. Subjects were given a visual demonstration of the required test postures, followed by consistent tactile feedback to guide appropriate test postures if required.

### Pair wise comparison positions

Comparisons of EMG activation were made in four paired conditions (Figure 1):

1. **Anterior Trunk Sway** vs. **Posterior Trunk Sway** was defined by the “Thorax Angle X” from the Full Body Plug-in Gait model. This is the position of the thorax relative to space in the sagittal plane. The Thorax Angle X from the Upright Standing posture for each subject was used as the reference angle. The Anterior Trunk Sway and Posterior Trunk Sway angles were defined as at least 15° anterior and posterior to the Upright Standing posture Thorax Angle X respectively. A positive value represents magnitude of anterior sway and a negative value represents magnitude of posterior sway.

2. **Left Trunk Shift** vs. **Right Trunk Shift** was defined by the “Thorax Angle Y”. This is the position of the thorax relative to space in the frontal plane. The Thorax Angle Y from the Upright Standing posture was used as the reference Thorax Angle. The Left Trunk Shift and Right Trunk Shift angles were defined as at least 10° left and right of the Upright Standing posture Thorax Angle Y respectively. A positive value represents magnitude of Left Trunk Shift and a negative value represents magnitude of Right Trunk Shift.

3. **Anterior Pelvic Rotation** vs. **Posterior Pelvic Rotation** was defined by the “Pelvis Angle X”. This is the position of the pelvis relative to space in the sagittal plane. The Pelvis Angle X from the Upright Standing posture was used as the reference Pelvis Angle. The Anterior Pelvic Rotation and Posterior Pelvic Rotation angles were defined as at least 5° anterior and posterior to the Upright Standing posture Pelvic Angle respectively. A positive value represents magnitude of Anterior Pelvic Rotation and a negative value represents magnitude of Posterior Pelvic Rotation.

4. **Lateral Pelvic Drop** vs. **Lateral Pelvic Raise** was defined by the “Pelvis Angle Y”. This is the position of the pelvis relative to space in the frontal plane, and the “Lateral Pelvis” makes reference to the subjects left hemi-pelvis, contra lateral to the loaded limb. The Pelvis Angle Y from the Upright Standing posture for each subject was used as the reference Pelvis Angle. The Lateral Pelvic Drop and Lateral Pelvic Raise angles were defined as at least 5° higher and lower of the Upright Standing posture Pelvis Angle respectively. A positive value represents magnitude of Lateral Pelvic Drop and a negative value represents magnitude of Lateral Pelvic Raise.

### Muscle activity

Surface EMG (using electrode placement as defined by Perotto [23]) of the following muscles were recorded: gluteus maximus; gluteus medius; TFL; semitendinosus; biceps femoris (long head); vastus lateralis; rectus femoris; and adductor longus.

EMG signals were recorded using integral dry reusable electrodes with an inter-electrode distance of 20 mm (Biometrics SX230, Gwent, UK). Low impedance between electrodes was obtained by abrading and cleaning the skin with emery paper and alcohol. Signals were recorded at a sampling frequency of 1000 Hz using Biometrics hardware (Biometrics DataLOG, Gwent, UK) and dedicated software. EMG signals were amplified and filtered (band pass 30 Hz – 500 Hz, gain = 1000) and muscle electrical activity was determined by calculating the mean value of the root mean square (RMS) over a stable four second period. A common earth electrode was placed over the wrist. Raw data were visually inspected for stability and consistency prior to selection of a stable four seconds of data for analysis.

EMG for each of the paired test postures was expressed as a percentage of the reference Upright Standing posture. We normalized EMG to Upright Standing representing a submaximal voluntary contraction (SubMVC) normalization method.

Six trials of each test posture were conducted with 30 seconds rest between each trial to limit the effects of fatigue. The order of test postures was selected randomly via computer generated randomization with the exception of Upright Standing, which was always performed first and formed the reference position from which the other test postures were then guided by the investigator.

Independent knee, hip, pelvis and trunk angles in the sagittal and frontal planes (Vicon Plug-in Gait model) were also monitored for consistency across trials for each test posture.

### Statistical analysis

All data were coded and analyzed using the SPSS statistical software v19.0 (SPSS inc., USA). In order to establish the reliability of the test posture angles and reliability of muscle activation in the reference upright posture and the eight test postures, intraclass correlation coefficient (ICC (2,1)) was computed [24]. Repeated measures ANOVA was performed along with associated F-tests to allow calculation of the Standard Error of the Measurement (SEM) and to determine if there were significant differences in muscle activation between each of the paired movements. An alpha level of p < 0.05 was set to determine significance.

## Results

### Kinematics

Pair wise comparisons of the four paired test postures demonstrated their validity as distinct postures based on large differences between criterion trunk or pelvic angles for each of the four paired postures. Table 1 shows all angles that displayed a significant difference between paired postures. Angles not mentioned experienced no significant change and therefore displayed consistency throughout testing.

**Table 1:** Mean changes in angles of interest during pair wise test posture comparisons

**Pair wise positions**	**Angles**	**Mean change (95% CI)**	**P value**
Anterior Trunk Sway vs. Posterior Trunk Sway	R Hip X	15° (12° - 18°)	<0.001
	R Pelvis X	9° (7° - 12°)	<0.001
	R Spine X	22° (19° - 26°)	<0.001
	R Thorax X*	**31° (28° - 34°)**	**<0.001**
Anterior Pelvic Rotation vs. Posterior Pelvic Rotation	R Hip X	16° (13° - 18°)	<0.001
	R Pelvis X*	**15° (13°- 17°)**	**<0.001**
	R Pelvis Y	1° (0° - 2°)	0.028
	R Spine X	−18° (−21 - -16°)	<0.001
	R Thorax X	−3° (−5° - -1°)	<0.001
Left Trunk Shift vs. Right Trunk Shift	R Pelvis Y	3° (1°- 4°)	<0.001
	R Thorax Y*	**26° (24° - 28°)**	**<0.001**
Pelvic Drop vs. Pelvic Raise	R Pelvis Y*	**14° (12–16)**	**<0.001**

The bold/* angles highlight the defining angle for each of the pair wise position.

### EMG

#### Anterior trunk sway vs. Posterior trunk sway

**Figure 2:**
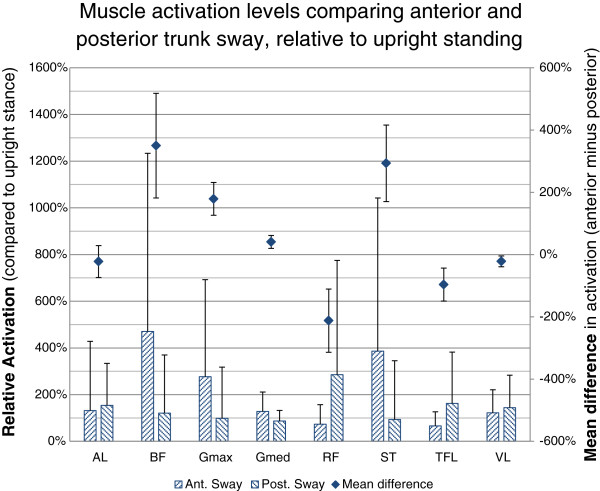
**Muscle activation levels in anterior trunk sway compared to posterior trunk sway.** Muscle activation levels are presented as the relative change in EMG to the reference Upright Standing (hatched bars) as well as the mean of the individual differences in activation (diamonds). Positive difference values indicate higher activation levels for the given muscle in anterior trunk sway, negative values represent increased activation levels in posterior trunk sway. The values are the difference relative to the activation level in upright stance. For example, semitendinosus activation is higher (293% of the level in upright stance) in **Anterior** Trunk Sway compared to Posterior Trunk Sway, whereas rectus femoris is activated more (212%) in **Posterior** Sway compared to Anterior Sway. The 95% CI are represented by the whiskers. Semitendinosus (ST); biceps femoris (BF) (long head); gluteus maximus (Gmax); rectus femoris (RF); vastus lateralis (VL); tensor fascia lata (TFL); gluteus medius (Gmed); and adductor longus (AL).

When comparing muscle activation in the Anterior Trunk Sway relative to Posterior Trunk Sway, the posterior sagittal plane muscles (semitendinosus [difference: +293%, 95 CI: 170% to 416%, p <0.001], biceps femoris [+350%, 182% to 518%, p <0.001], gluteus maximus [+178%, 126% to 231%, p <0.001]) all markedly increased in activation while the anterior sagittal plane muscles (rectus femoris [−212%, 111% to −314%, p <0.001], vastus lateralis [−220%, −5% to −39%, p = 0.016], TFL [−96%, −43% to −149%, p = 0.001]) showed decreased activation levels (Figure 2).

#### Left trunk shift vs. Right trunk shift

**Figure 3:**
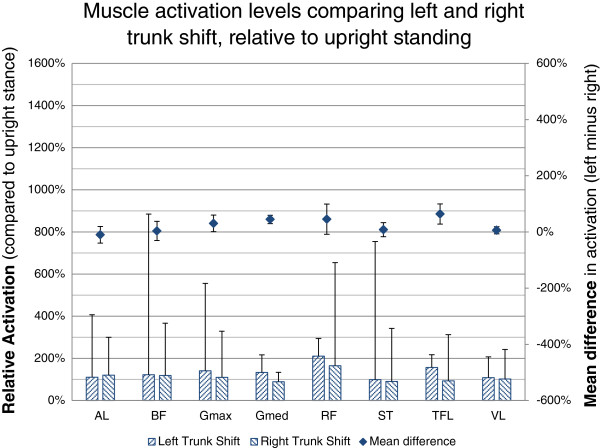
**Muscle activation levels in left trunk shift compared to right trunk shift.** Muscle activation levels are presented as the relative change in EMG to the reference Upright Standing (hatched bars) as well as the mean of the individual differences in activation (diamonds). Positive difference values indicate higher activation levels for the given muscle in Left Trunk Shift, negative values represent increased activation levels in Right Trunk Shift. The values are the difference relative to the activation level in upright stance. 95% CI are represented by the whiskers. Semitendinosus (ST); biceps femoris (BF) (long head); gluteus maximus (Gmax); rectus femoris (RF); vastus lateralis (VL); tensor fascia lata (TFL); gluteus medius (Gmed); and adductor longus (AL).

When comparing muscle activation of Left Trunk Shift relative to Right Trunk Shift, the lateral hip abductors (gluteus medius [+45%, 30% to 59%, p < 0.001] and, TFL [+28%, 64% to 100%, p = 0.001]) showed increased activation as did gluteus maximus (+31%, 1% to 60%, p = 0.043). There were no significant differences found in the other muscles (Figure 3).

#### Anterior pelvic rotation vs. Posterior pelvic rotation

**Figure 4:**
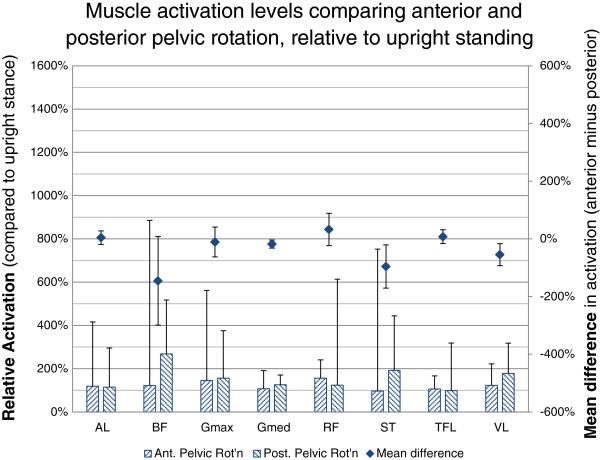
**Muscle activation levels in anterior pelvic rotation compared to posterior pelvic rotation.** Muscle activation levels are presented as the relative change in EMG to the reference Upright Standing (hatched bars) as well as the mean of the individual differences in activation (diamonds). Positive difference values indicate higher activation levels for the given muscle in Anterior Pelvic Rotation, negative values represent increased activation levels in Posterior Pelvic Rotation. The values are the difference relative to the activation level in upright stance. 95% CI are represented by the whiskers. Semitendinosus (ST); biceps femoris (BF) (long head); gluteus maximus (Gmax); rectus femoris (RF); vastus lateralis (VL); tensor fascia lata (TFL); gluteus medius (Gmed); and adductor longus (AL).

When comparing muscle activation of Anterior Pelvic Rotation relative to Posterior Pelvic Rotation, the vastus lateralis showed a significant decrease in muscle activation (−56%, −17% to −55%, p = 0.007) as did the semitendinosus (−171%, −21% to −96%, p = 0.015) and gluteus medius (−178%, −4% to −32%, p = 0.015). There were no significant differences found in the other muscles (Figure 4).

#### Lateral pelvic drop vs. Lateral pelvic raise

**Figure 5:**
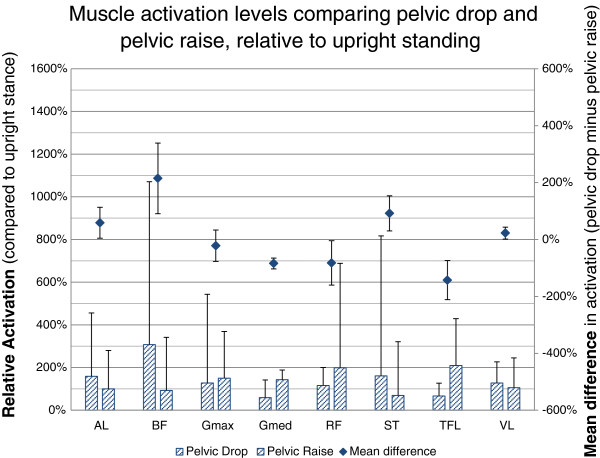
**Muscle activation levels in pelvic drop compared to pelvic raise.** Muscle activation levels are presented as the relative change in EMG to the reference Upright Standing (hatched bars) as well as the mean of the individual differences in activation (diamonds). Positive difference values indicate higher activation levels for the given muscle in Pelvic Drop, negative values represent increased activation levels in Pelvic Raise. The values are the difference relative to the activation level in upright stance. 95% CI are represented by the whiskers. Semitendinosus (ST); biceps femoris (BF) (long head); gluteus maximus (Gmax); rectus femoris (RF); vastus lateralis (VL); tensor fascia lata (TFL); gluteus medius (Gmed); and adductor longus (AL).

This data is based on 20 subjects as two subjects were unable to adopt the Lateral Pelvic Drop position. When comparing muscle activation of Lateral Pelvic Drop relative to Lateral Pelvic Raise, the lateral hip abductors (gluteus medius [−84%, −65% to −104%, p < 0.001], and TFL [−143%, −74% to −212%, p < 0.001]) showed decreased activation as did the rectus femoris (−82%, −4% to −160%, p = 0.04). The hamstring group (semitendinosus [+92%, 30% to 154%, p = 0.006] and biceps femoris [+214%, 91% to 339%, p = 0.002]) showed increased activation as did the adductor longus (+58%, 4% to 113%, p = 0.036) and vastus lateralis (+22%, 2% to 43%, p = 0.037) (Figure 5).

### Reliability of test postures and measures

#### Kinematic reliability

Intraclass correlation coefficient values for each of the seven joint angles across each of the nine test postures over six trials ranged from 0.54 to 0.95 (p < 0.001) (see Additional file 1).

The ICC’s of the kinematic measures showed reliability in excess of 0.75 except for Thorax Y (frontal plane) with a mean ICC of 0.54. SEM data for each of the test positions are presented in Additional file 1.

#### EMG reliability

The ICC values for the 72 possible values (eight muscles across nine positions) ranged from 0.29-0.97 (p < 0.001). The majority of muscles in all positions, for all subjects over six trials showed ICC values ranging from 0.75 to 0.97 with 16 exceptions. Adductor longus displayed decreased reliability during: Upright Standing; all pelvic positions; and Left Trunk Shift with mean ICC’s of 0.29-0.74. Semitendinosis activity was also less repeatable with mean ICC’s 0.36-0.69 during the positions of Upright Standing, Lateral Pelvic Raise, Posterior Pelvic Rotation, Left and Right Trunk Shift, and Posterior Trunk Sway. Biceps femoris activity during Posterior Trunk Sway and Right Trunk Shift had mean ICC’s 0.51-0.73. Vastus lateralis during Left Trunk Shift had a mean ICC of 0.66. Tensor Fascia Lata during Right Trunk Shift had a mean ICC of 0.74 (see Additional file 2).

## Discussion

The results of this study demonstrated that changes in trunk and pelvic posture in single leg stance strongly influence the levels of activation of different muscles of the hip and thigh. The magnitude of these changes support that positioning of the trunk and pelvis relative to the hips is important.

### Trunk posture changes

#### Anterior trunk sway vs. Posterior trunk sway

There was a clear pattern of activation of the posterior hip muscles and a concurrent de-activation of the anterior hip muscles as the trunk shifted anterior to the pelvis. O’Sullivan et al. [15] reported a consistent pattern of activation of the posterior trunk muscles and de-activation of the upper anterior abdominal wall with the same body position change. Similar findings for the hip muscles have previously been reported by Wang et al., in double leg stance [18]. Changes in the activation of the sagittal plane muscles such as rectus femoris and the hamstrings between anterior and posterior trunk sway were very large, where the magnitude of the changes were two- to three-fold. These findings support that altering the sagittal position of the trunk in relation to the hip during single leg stance has a powerful influence on hip and thigh muscles that control sagittal plane movement.

#### Left trunk shift vs. Right trunk shift

For the lateral trunk shift condition, increased activation of the hip abductor muscles (gluteus maximus, gluteus medius and TFL) was demonstrated with the Left Trunk Shift position when standing on the right leg. These findings support that frontal plane movement of the trunk away from the weightbearing leg results in a greater demand on the hip abductor muscles to maintain balance. We also hypothesized we would observe an increase in adductor longus activation in Right Trunk Shift posture for the same reason, however this was not observed. The absence of this finding was reflected in the large variability in EMG responses observed in this muscle during Right Trunk Shift. Visual graphical inspection of the individual subject activation patterns highlighted some subjects had increased levels of adductor longus activity that was above the Upright Standing position in either Left or Right Trunk Shift. It remains to be seen whether these variations are distributed evenly, or clustered in populations of high and low activation, and this will not likely be resolved until larger numbers of subjects are examined. This observation warrants further investigation in clinical populations to determine whether these findings show any relationship to the presence of adductor-related injury [25].

### Pelvic posture changes

#### Anterior pelvic rotation vs. Posterior pelvic rotation

The differences in muscle activation when the postural adjustment was initiated via the pelvis in the sagittal plane are more difficult to interpret. It was noted that there was significant variability in terms of the direction of the change in muscle activation in this pair wise comparison compared to the other conditions for TFL, gluteus maximus, rectus femoris and the hamstring muscles. This variability suggests a range of different motor control strategies for the same task in different individuals.

#### Lateral pelvic drop vs. Lateral pelvic raise

In the Lateral Pelvic Drop relative to Lateral Pelvic Raise position, there was a clear pattern of reduced activation of the hip abductor muscles (TFL and gluteus medius) and rectus femoris with a concurrent increase in activation of the hamstrings, adductor longus and vastus lateralis muscles. These findings suggest a shift in activation away from the hip abductors in the ‘Trendelenberg’ posture. The Trendelenburg posture has been related with a number of clinical presentations [14] and is thought to be a relatively passive position requiring little hip abductor muscle activation. Our results support this clinical interpretation for the hip abductor muscles (gluteus medius and TFL), however the concurrent increased activation of other muscles may have clinical implications in populations such as hamstring and groin injury. By establishing the presence of consistent muscle activation patterns in pain free subjects, the motor strategies of such clinical populations can now be investigated to inform injury prevention and rehabilitation considerations.

In contrast to the Trendelenburg position, Lateral Pelvic Raise, required greater activity in the hip abductor muscles to maintain the contralateral pelvis elevated, which has been reported previously [26]. These findings may have implications for functional retraining of frontal plane muscles by focusing on simple changes to frontal plane pelvic posture during functional tasks.

Normalising EMG to a single leg stance reference posture as a Sub-maximal Isometric Voluntary Contraction (SubMIVC) has previously been documented by Norcross et al. [20], with similarly small variations reported. Although a limitation with using a SubMIVC method can be finding equivalent submaximal loads for different muscles [21,22], SubMIVC has been shown to be reliable both when assessing low level muscle activity [22,23] and also in a static single leg stance position [20], which closely reflects our study design.

Adductor longus and semitendinosus displayed poorer reliability which may explain why the expected change in EMG activation in our frontal plane test positions for adductor longus and semitendinosus were not observed. The variability displayed in activation of the adductor longus muscle is of clinical interest. During sporting activity, adductor related groin pain is a significant burden comprising approximately 8 – 16% in footballers [24-26]. The variability in activation levels of the adductor longus displayed in this normal healthy population of active males suggests this may be an avenue for examination in populations where adductor-related groin pain is of interest.

### Limitations

The findings of this study only apply to asymptomatic males, therefore we cannot make any conclusions about females, the very young, older, or clinical populations. Further, we looked at activation of superficial muscles in single plane directions. The assessment of deeper muscles and muscles in a range of multi-directional functional trunk and pelvic postures may be important. We are unable to recommend the use of Upright Standing in single leg stance as a SubMVC method to normalise EMG to if the muscles adductor longus and/or semitendinosus are the intended muscles of investigation.

To validate the Upright Standing position as the position for EMG normalization and therefore our reference posture, reliability of subject positioning needs to be demonstrated. The ICC’s of the kinematic measures showed reliability in excess of 0.75 except for Thorax Y (frontal plane) with a mean ICC of 0.54. This ICC needs to be considered in light of the magnitude of the values and the SEM of 1.4° which we contend is clinically trivial variability. Throughout the pair wise test positions, the mean ICC’s of the majority of angles showed reliability over 0.70 across the six trials. Thorax Y (frontal plane) during Lateral Pelvic Drop and Lateral Pelvic Raise were the exceptions with ICC’s (SEM) of 0.54 (2.8°), and 0.63 (2.1°) respectively. Similar to the Upright Stance posture, the SEM values for Thorax Y angle still suggest clinical utility.

## Conclusions

This study established patterns of hip and thigh muscle activation during common functional single leg loading postures. Displacement of the trunk in the sagittal plane influences activation of muscles that control sagittal plane movement. Adjustments of trunk and pelvis posture in the frontal plane primarily influences activation of muscles that control frontal plane movement. The magnitude of these changes between paired test postures support that positioning of the trunk and pelvis relative to the hips is important. These findings can be now compared in symptomatic populations as a possible mechanism for injury and have implications for exercise rehabilitation of functional single leg loading tasks.

## Abbreviations

EMG: Electromyography; BMI: Body mass index; ICC: Intraclass correlation coefficient; SubMIVC: Sub-maximal Isometric Voluntary Contraction; TFL: Tensor fascia lata.

## Competing interests

The author(s) declare that they have no competing interests.

## Authors’ contributions

SP contributed to the conception and design, was involved in drafting the manuscript, revising it critically and giving final approval of the version to be published, was involved with the acquisition/analysis and interpretation of data. PO contributed to the conception and design, was involved in drafting the manuscript, revising it critically and giving final approval of the version to be published, was involved with the analysis and interpretation of data. TM contributed to the conception and design, was involved in drafting the manuscript, revising it critically and giving final approval of the version to be published, was involved with the acquisition/analysis and interpretation of data. RW was involved in drafting the manuscript, revising it critically and giving final approval of the version to be published, was involved with the analysis and interpretation of data. BW contributed substantially to acquisition of data, analysis and interpretation of data. SR contributed substantially to the design, analysis and interpretation of data. AF contributed substantially to the analysis and interpretation of data. All authors read and approved the final manuscript.

## Pre-publication history

The pre-publication history for this paper can be accessed here:

http://www.biomedcentral.com/2052-1847/6/13/prepub

## Supplementary Material

Additional file 1 Kinematic reliability. Click here for file

Additional file 2 EMG reliability. Click here for file
