# Supplementary material for: The influence of changes in trunk and pelvic posture during single leg standing on hip and thigh muscle activation in a pain free population
Source: BMC Sports Sci Med Rehabil. 2014 Mar 27;6:13. doi: 10.1186/2052-1847-6-13 (PMC4022336; doi:10.1186/2052-1847-6-13)
Supplement: Additional file 1 — Kinematic reliability. [file 2052-1847-6-13-S1.docx]

Additional File 1 (Kinematic reliability)

|  |  |  |  |
| --- | --- | --- | --- |
| **Upright Standing** | | | |
| **Angle** | **Mean° ± SD (SEM)** | **ICC (95%CI)** | **p-value** |
| **R Hip X** | 12.2±5.8 (2.0) | 0.89(0.81-0.95) | <0.001 |
| **R Knee X** | 9.0±5.5 (2.0) | 0.88(0.8-0.94) | <0.001 |
| **R Pelvis X** | 12.0±3.9 (1.1) | 0.91(0.85-0.96) | <0.001 |
| **R Pelvis Y** | -1.1±2.8 (1.1) | 0.87(0.79-0.94) | <0.001 |
| **R Spine X** | -14.8±5.4 (1.5) | 0.93(0.88-0.97) | <0.001 |
| **R Thorax X** | -2.7±3.9 (1.1) | 0.91(0.85-0.96 | <0.001 |
| **R Thorax Y** | -1.8±1.6 (1.4) | 0.54(0.36-0.73) | <0.001 |
|  |  |  |  |
| **Anterior Trunk Sway** | | | |
| **Angle** | **Mean° ± SD (SEM)** | **ICC (95%CI)** | **p-value** |
| **R Hip X** | 21.9±5.7 (2.8) | 0.81(0.69-0.90) | <0.001 |
| **R Knee X** | 13.3±5.8 (2.4) | 0.85(0.75-0.92) | <0.001 |
| **R Pelvis X** | 15.7±4.4 (1.9) | 0.84(0.74-0.92) | <0.001 |
| **R Pelvis Y** | 0.2±3.2 (1.3) | 0.85(0.75-0.92) | <0.001 |
| **R Spine X** | 1.7±8.6 (2.6) | 0.93(0.87-0.96) | <0.001 |
| **R Thorax X** | 17.4±6.7 (2.5) | 0.89(0.80-0.95) | <0.001 |
| **R Thorax Y** | -1.7±2.5 (1.2) | 0.80(0.68-0.90) | <0.001 |
|  |  |  |  |
| **Posterior Trunk Sway** | | | |
| **Angle** | **Mean° ± SD (SEM)** | **ICC (95%CI)** | **p-value** |
| **R Hip X** | 6.9±6.8 (2.7) | 0.86(0.77-0.93) | <0.001 |
| **R Knee X** | 11.7±5.6 (2.0) | 0.88(0.79-0.94) | <0.001 |
| **R Pelvis X** | 6.6±4.6 (1.8) | 0.86(0.76-0.93) | <0.001 |
| **R Pelvis Y** | 0.1±2.6 (1.2) | 0.82(0.71-0.91) | <0.001 |
| **R Spine X** | -20.5±7.3 (1.8) | 0.94(0.89-0.97) | <0.001 |
| **R Thorax X** | -13.9±4.2 (1.7) | 0.84(0.74-0.92) | <0.001 |
| **R Thorax Y** | -2.1±2.0 (1.1) | 0.72(0.56-0.85) | <0.001 |
|  |  |  |  |
| **Anterior Pelvic Rotation** | | | |
| **Angle** | **Mean° ± SD (SEM)** | **ICC (95%CI)** | **p-value** |
| **R Hip X** | 21.8±6.0 (2.5) | 0.83(0.72-0.92) | <0.001 |
| **R Knee X** | 12.0±5.6 (2.4) | 0.85(0.74-0.92) | <0.001 |
| **R Pelvis X** | 18.9±3.2 (1.8) | 0.76(0.62-0.87) | <0.001 |
| **R Pelvis Y** | -0.3±2.7 (1.3) | 0.82(0.71-0.91) | <0.001 |
| **R Spine X** | -23.5±5.7 (1.9) | 0.90(0.83-0.95) | <0.001 |
| **R Thorax X** | -4.6±4.3 (1.7) | 0.87(0.78-0.94) | <0.001 |
| **R Thorax Y** | -2.2±2.5 (1.0) | 0.87(0.78-0.94) | <0.001 |
|  |  |  |  |
| **Posterior Pelvic Rotation** | | | |
| **Angle** | **Mean° ± SD (SEM)** | **ICC (95%CI)** | **p-value** |
| **R Hip X** | 6.4±6.2 (2.1) | 0.89(0.81-0.94) | <0.001 |
| **R Knee X** | 13.0±5.4 (1.9) | 0.89(0.81-0.94) | <0.001 |
| **R Pelvis X** | 3.8±4.4 (1.6) | 0.87(0.78-0.93) | <0.001 |
| **R Pelvis Y** | -1.4±2.5 (1.1) | 0.84(0.73-0.92) | <0.001 |
| **R Spine X** | -5.3±6.9 (2.1) | 0.91(0.85-0.96) | <0.001 |
| **R Thorax X** | -1.5±4.4 (1.6) | 0.89(0.81-0.95) | <0.001 |
| **R Thorax Y** | -1.3±2.3 (1.2) | 0.79(0.66-0.89) | <0.001 |
|  |  |  |  |
| **Left Trunk Shift** | | | |
| **Angle** | **Mean° ± SD (SEM)** | **ICC (95%CI)** | **p-value** |
| **R Hip X** | 14.3±5.8 (1.7) | 0.90(0.82-0.95) | <0.001 |
| **R Knee X** | 11.1±5.9 (1.9) | 0.90(0.83-0.95) | <0.001 |
| **R Pelvis X** | 12.6±4.3 (1.3) | 0.90(0.83-0.95) | <0.001 |
| **R Pelvis Y** | 0.3±3.1 (1.5) | 0.81(0.70-0.90) | <0.001 |
| **R Spine X** | -15.8±7.2 (1.6) | 0.95(0.91-0.97) | <0.001 |
| **R Thorax X** | -3.6±4.1 (1.3) | 0.90(0.83-0.95) | <0.001 |
| **R Thorax Y** | 10.3±3.3 (2.1) | 0.71(0.56-0.85) | <0.001 |
|  |  |  |  |
| **Right Trunk Shift** | | | |
| **Angle** | **Mean° ± SD (SEM)** | **ICC (95%CI)** | **p-value** |
| **R Hip X** | 13.9±5.7 (2.2) | 0.84(0.73-0.92) | <0.001 |
| **R Knee X** | 10.0±5.4 (2.2) | 0.84(0.73-0.92) | <0.001 |
| **R Pelvis X** | 12.7±4.3 (1.4) | 0.89(0.82-0.95) | <0.001 |
| **R Pelvis Y** | -2.2±3.1 (1.9) | 0.73(0.58-0.85) | <0.001 |
| **R Spine X** | -15.7±7.0 (1.7) | 0.94(0.90-0.97) | <0.001 |
| **R Thorax X** | -2.9±4.4 (1.3) | 0.93(0.87-0.97) | <0.001 |
| **R Thorax Y** | -15.6±3.5 (2.2) | 0.70(0.55-0.84) | <0.001 |
|  |  |  |  |
| **Lateral Pelvic Drop** | | | |
| **Angle** | **Mean° ± SD (SEM)** | **ICC (95%CI)** | **p-value** |
| **R Hip X** | 15.5±7.0 (1.7) | 0.93(0.88-0.97) | <0.001 |
| **R Knee X** | 10.7±7.7 (1.8) | 0.95(0.91-0.98) | <0.001 |
| **R Pelvis X** | 13.7±4.4 (1.2) | 0.91(0.84-0.96) | <0.001 |
| **R Pelvis Y** | 6.4±2.9 (1.1) | 0.84(0.72-0.92) | <0.001 |
| **R Spine X** | -16.5±6.7 (4.0) | 0.74(0.58-0.86) | <0.001 |
| **R Thorax X** | -4.0±4.8 (2.1) | 0.83(0.72-0.92) | <0.001 |
| **R Thorax Y** | -1.7±3.1 (2.8) | 0.54(0.36-0.74) | <0.001 |
|  |  |  |  |
| **Lateral Pelvic Raise** | | | |
| **Angle** | **Mean° ± SD (SEM)** | **ICC (95%CI)** | **p-value** |
| **R Hip X** | 15.6±7.2 (1.6) | 0.95(0.92-0.98) | <0.001 |
| **R Knee X** | 9.9±6.0 (2.2) | 0.87(0.79-0.94) | <0.001 |
| **R Pelvis X** | 14.6±4.5 (1.3) | 0.92(0.86-0.96) | <0.001 |
| **R Pelvis Y** | -7.6±2.7 (1.5) | 0.75(0.61-0.87) | <0.001 |
| **R Spine X** | -17.8±7.0 (1.6) | 0.95(0.90-0.97) | <0.001 |
| **R Thorax X** | -3.8±4.4 (1.3) | 0.93(0.87-0.96) | <0.001 |
| **R Thorax Y** | -2.1±2.7 (2.1) | 0.63(0.45-0.79) | <0.001 |
