# Supplementary material for: The influence of changes in trunk and pelvic posture during single leg standing on hip and thigh muscle activation in a pain free population
Source: BMC Sports Sci Med Rehabil. 2014 Mar 27;6:13. doi: 10.1186/2052-1847-6-13 (PMC4022336; doi:10.1186/2052-1847-6-13)
Supplement: Additional file 2 — EMG reliability. [file 2052-1847-6-13-S2.docx]

Additional file 2 (EMG reliability)

| **Upright Standing** | | | |
| --- | --- | --- | --- |
| **Muscle** | **Mean ± SD (SEM)** | **ICC (95%CI)** | **p-value** |
| **AL** | 4.87 ± 1.80(2.46) | 0.29(011-0.52) | <0.001 |
| **BF** | 18.76 ± 22.77(7.93) | 0.90(0.82-0.95) | <0.001 |
| **Gmax** | 9.25 ± 7.12(2.65) | 0.87(0.77-0.93) | <0.001 |
| **Gmed** | 25.08 ± 18.34(3.15) | 0.97(0.94-0.98) | <0.001 |
| **RF** | 26.28 ± 27.93(10.03) | 0.88(0.79-0.94) | <0.001 |
| **ST** | 17.71 ± 12.68(9.41) | 0.64(0.47-0.80) | <0.001 |
| **TFL** | 47.96 ± 31.09(11.78) | 0.89(0.80-0.94) | <0.001 |
| **VL** | 42.71 ± 21.39(10.24) | 0.80(0.68-0.90) | <0.001 |
|  |  |  |  |
|  |  |  |  |
| **Anterior Trunk Sway** | | | |
| **Muscle** | **Mean ± SD (SEM)** | **ICC (95%CI)** | **p-value** |
| **AL** | 5.40 ± 4.62(0.84) | 0.97(0.95-0.99) | <0.001 |
| **BF** | 50.19 ± 30.29(12.34) | 0.87(0.77-0.93) | <0.001 |
| **Gmax** | 20.52 ± 12.08(3.29) | 0.93(0.88-0.97) | <0.001 |
| **Gmed** | 29.99 ± 18.40(4.63) | 0.96(0.93-0.98) | <0.001 |
| **RF** | 12.71 ± 12.93(4.48) | 0.94(0.88-0.97) | <0.001 |
| **ST** | 45.88 ± 22.37(7.95) | 0.88(0.80-0.94) | <0.001 |
| **TFL** | 26.62 ± 15.79(5.48) | 0.87(0.78-0.94) | <0.001 |
| **VL** | 47.19 ± 21.45(10.07) | 0.84(0.74-0.92) | <0.001 |
|  |  |  |  |
|  |  |  |  |
| **Posterior Trunk Sway** | | | |
| **Muscle** | **Mean ± SD (SEM)** | **ICC (95%CI)** | **p-value** |
| **AL** | 6.93 ± 4.26(1.04) | 0.94(0.89-0.97) | <0.001 |
| **BF** | 10.76 ± 6.07(6.14) | 0.51(0.32-0.71) | <0.001 |
| **Gmax** | 6.87 ± 5.44(1.50) | 0.92(0.86-0.96) | <0.001 |
| **Gmed** | 22.43 ± 17.42(5.23) | 0.96(0.93-0.98) | <0.001 |
| **RF** | 49.59 ± 44.66(15.79) | 0.90(0.83-0.95) | <0.001 |
| **ST** | 9.04 ± 7.36(8.13) | 0.36(0.18-0.59) | <0.001 |
| **TFL** | 71.80 ± 57.65(23.25) | 0.85(0.75-0.92) | <0.001 |
| **VL** | 56.65 ± 29.79(13.30) | 0.83(0.72-0.92) | <0.001 |

|  |  |  |  |
| --- | --- | --- | --- |
|  |  |  |  |
| **Anterior Pelvic Rotation** | | | |
| **Muscle** | **Mean ± SD (SEM)** | **ICC (95%CI)** | **p-value** |
| **AL** | 5.36 ± 2.09(1.44) | 0.74(0.60-0.87) | <0.001 |
| **BF** | 17.01 ± 16.37(7.57) | 0.83(0.71-0.91) | <0.001 |
| **Gmax** | 11.12 ± 5.60(2.15) | 0.88(0.80-0.94) | <0.001 |
| **Gmed** | 26.84 ± 25.66(3.79) | 0.98(0.96-0.99) | <0.001 |
| **RF** | 29.22 ± 26.59(11.16) | 0.84(0.73-0.92) | <0.001 |
| **ST** | 13.87 ± 10.61(4.87) | 0.82(0.70-0.91) | <0.001 |
| **TFL** | 47.50 ± 29.32(12.03) | 0.84(0.74-0.92) | <0.001 |
| **VL** | 51.15 ± 28.21(7.39) | 0.93(0.87-0.97) | <0.001 |
|  |  |  |  |
|  |  |  |  |
| **Posterior Pelvic Rotation** | | | |
| **Muscle** | **Mean ± SD (SEM)** | **ICC (95%CI)** | **p-value** |
| **AL** | 5.10 ± 1.37(0.94) | 0.63(0.46-0.80) | <0.001 |
| **BF** | 33.66 ± 37.26(13.75) | 0.86(0.77-0.93) | <0.001 |
| **Gmax** | 13.58 ± 13.49(3.37) | 0.93(0.88-0.97) | <0.001 |
| **Gmed** | 31.01 ± 24.37(10.69) | 0.88(0.79-0.94) | <0.001 |
| **RF** | 21.82 ± 16.22(8.16) | 0.82(0.71-0.91) | <0.001 |
| **ST** | 28.41 ± 21.47(14.37) | 0.69(0.53-0.83) | <0.001 |
| **TFL** | 41.30 ± 30.19(14.68) | 0.79(0.66-0.89) | <0.001 |
| **VL** | 66.16 ± 30.07(12.54) | 0.86(0.76-0.93) | <0.001 |
|  |  |  |  |
|  |  |  |  |
| **Left Trunk Shift** | | | |
| **Muscle** | **Mean ± SD (SEM)** | **ICC (95%CI)** | **p-value** |
| **AL** | 5.01 ± 1.49(1.42) | 0.51(0.32-0.71) | <0.001 |
| **BF** | 17.38 ± 16.93(8.58) | 0.76(0.62-0.88) | <0.001 |
| **Gmax** | 11.74 ± 9.20(2.86) | 0.91(0.83-0.95) | <0.001 |
| **Gmed** | 32.95 ± 23.73(4.75) | 0.96(0.93-0.98) | <0.001 |
| **RF** | 38.81 ± 31.72(14.42) | 0.83(0.72-0.92) | <0.001 |
| **ST** | 16.35 ± 14.26(12.46) | 0.53(0.34-0.73) | <0.001 |
| **TFL** | 67.12 ± 39.60(19.63) | 0.80(0.67-0.90) | <0.001 |
| **VL** | 43.82 ± 20.23(13.64) | 0.66(0.49-0.81) | <0.001 |
|  |  |  |  |

|  |  |  |  |
| --- | --- | --- | --- |
| **Right Trunk Shift** | | | |
| **Muscle** | **Mean ± SD (SEM)** | **ICC (95%CI)** | **p-value** |
| **AL** | 5.67 ± 4.41(2.09) | 0.90(0.82-0.95) | <0.001 |
| **BF** | 16.68 ± 17.60(10.13) | 0.73(0.57-0.85) | <0.001 |
| **Gmax** | 8.87 ± 7.35(2.35) | 0.94(0.90-0.97) | <0.001 |
| **Gmed** | 21.73 ± 16.52(4.26) | 0.94(0.89-0.97) | <0.001 |
| **RF** | 28.04 ± 20.93(7.69) | 0.86(0.77-0.93) | <0.001 |
| **ST** | 12.95 ± 9.00(8.07) | 0.47(0.28-0.68) | <0.001 |
| **TFL** | 41.70 ± 25.84(13.81) | 0.74(0.59-0.87) | <0.001 |
| **VL** | 42.64 ± 20.76(8.82) | 0.86(0.77-0.93) | <0.001 |
|  |  |  |  |
|  |  |  |  |
| **Lateral Pelvic Drop** | | | |
| **Muscle** | **Mean ± SD (SEM)** | **ICC (95%CI)** | **p-value** |
| **AL** | 6.79 ± 4.66(3.21) | 0.67(0.49-0.83) | <0.001 |
| **BF** | 34.48 ± 28.88(11.57) | 0.84(0.72-0.92) | <0.001 |
| **Gmax** | 8.64 ± 4.91(2.51) | 0.82(0.69-0.91) | <0.001 |
| **Gmed** | 12.70 ± 6.87(3.19) | 0.83(0.70-0.92) | <0.001 |
| **RF** | 17.85 ± 14.87(6.89) | 0.80(0.67-0.90) | <0.001 |
| **ST** | 20.86 ± 15.04(7.94) | 0.76(0.60-0.88) | <0.001 |
| **TFL** | 24.08 ± 12.63(6.07) | 0.80(0.66-0.90) | <0.001 |
| **VL** | 52.40 ± 37.57(13.24) | 0.87(0.77-0.94) | <0.001 |
|  |  |  |  |
|  |  |  |  |
| **Lateral Pelvic Raise** | | | |
| **Muscle** | **Mean ± SD (SEM)** | **ICC (95%CI)** | **p-value** |
| **AL** | 4.55 ± 1.13(0.79) | 0.61(0.42-0.78) | <0.001 |
| **BF** | 12.45 ± 12.57(6.62) | 0.81(0.69-0.90) | <0.001 |
| **Gmax** | 14.61 ± 14.62(3.54) | 0.95(0.90-0.97) | <0.001 |
| **Gmed** | 37.12 ± 35.23(4.41) | 0.99(0.98-0.99) | <0.001 |
| **RF** | 34.54 ± 27.19(12.82) | 0.78(0.65-0.89) | <0.001 |
| **ST** | 10.10 ± 8.22(5.78) | 0.63(0.45-0.79) | <0.001 |
| **TFL** | 82.17 ± 48.92(24.29) | 0.82(0.70-0.91) | <0.001 |
| **VL** | 41.77 ± 20.64(10.03) | 0.79(0.66-0.89) | <0.001 |
